# Discrete Element Modelling of Cold Crushing Tests Considering Various Interface Property Distributions in Ordinary Refractory Ceramics

**DOI:** 10.3390/ma15217650

**Published:** 2022-10-31

**Authors:** Weiliang Du, Shengli Jin

**Affiliations:** Department Mineral Resources Engineering, Montanuniversitaet Leoben, Peter-Tunner Strasse 5, 8700 Leoben, Austria

**Keywords:** discrete element method, interface property, heterogeneity, cold crushing test, fracture event, ordinary refractory ceramics

## Abstract

The microstructures and local properties of ordinary refractory ceramic materials are heterogeneous and play a role in the fracture behavior of ordinary refractory ceramic materials. It is important to consider them in numerical modeling. Herein, the discrete element (DE) method was applied to determine the influences of heterogeneity of ordinary refractory ceramic materials by applying statistically distributed interface properties (uniform, Weibull), as opposed to constant interface properties, among the elements. Uniaxial cold crushing tests were performed as a case study. A reasonable loading strain rate for receiving quasi-static loading conditions and computation efficiency was evaluated. The loading wall displacement was recorded to present the stress–strain curves of cold crushing tests. Furthermore, the effects of the interface property distributions on the load/displacement curve, fracture energy, cold crushing strength, and fracture events were investigated. The results reveal that the DE method is a promising method for visualizing and quantifying the post–peak fracture process and crack events in ordinary refractory ceramics. Different interface property distributions contribute to significant variances in the load/displacement curve shape and fracture pattern. The heterogeneity of ordinary refractory ceramics can be further determined by comparing the experimental curves and fracture propagation along with an inverse identification approach.

## 1. Introduction

Ordinary refractory ceramic materials are composed of various types of aggregates and fines. With a defined recipe and raw material resources, manufacturing processes, such as mixing, shaping, and thermal treatment, contribute to the heterogeneity in the microstructure and mechanical properties [1,2]. Dahlem et al. [3,4,5] investigated the factors influencing measured physical properties in a laboratory. The results revealed that the drilling direction plays a vital role in measuring the cold crushing strength and refractoriness under a load. Samadi et al. [6] reported that the creep behavior and Young’s modulus of an alumina spinel specimen vary with the sample drilling positions and directions. In order to account for the inhomogeneity of a magnesia spinel material, Dai et al. [7] considered a Weibull distribution of the mechanical properties and simulated the fracture process propagation during wedge splitting testing using a finite element (FE) method. The irreversible strain field from the FE simulation reasonably reproduced the crack propagation observed in the digital image correlation in the laboratory.

The discrete element (DE) method is a promising method for modeling geomaterials on a particle size scale [8,9,10,11,12,13,14]. This method was developed by Cundall and Strack [15] 40 years ago. With this method, rigid elements, such as circular disks, balls, and clumps, are regarded as the basic units; Newton’s second law determines the motion of each element caused by unbalanced contact forces, and the contact force is updated according to the force–displacement law [16]. DE simulations are widely used in the mechanical research of rocks [17], soils [18], concretes [19], and other granular materials [20,21,22], while DE simulations are still at the beginning in the field of ordinary refractory ceramics. Weiliang et al. [23] studied the influence of minimum element size on DE simulations of ordinary refractory ceramics. Asadi et al. [24] considered the influence of pre-existing microcracks on the thermal shock resistance of refractory ceramics.

The inhomogeneity of the geomaterial microstructure can be considered in the DE modeling. For instance, Caulk [25] studied a rock using cathodoluminescence image analysis and applied a Weibull distribution of interface strength to describe its heterogeneity during three-point bending tests using a DE method simulation. Nitka and Tejchman [26] considered a three-phase microstructure of concrete and defined different sets of interfacial properties for different phases. Wang et al. [27] reported the microstructure of concrete, with elements representing aggregates and mortar. The tensile strength and fracture energy between the aggregate and mortar was found to be weaker than those between the aggregates. Asadi et al. [28] simulated a wedge-splitting testing procedure for a material considering the Weibull distributed tensile strength, whereas the other mechanical parameters were homogeneous.

On the one hand, as aforementioned, the microstructure and local properties of ordinary refractory ceramic materials are heterogeneous. Thus, this must be considered in the modeling to obtain reasonable results. As an alternative method to finite element methods, DE methods can describe the microstructure features straightforwardly and explicitly at the grain level. Furthermore, constant interface properties are of ordinary refractory ceramics commonly considered in publications [25,26,27,28]. In contrast, this study considered a set of interface properties constituting a linear parallel bond model to be statistically distributed with the Weibull function or uniform function in defined value ranges. Herein, the case with a constant interface property distribution was regarded as the reference, and the cold crushing test was applied as a case study.

On the other hand, computational efficiency is always a haunting problem in DE applications. With a given configuration of interface and element properties, the critical time step is fixed, which is usually substantially small. One must consider the computation resources and resulting accuracy with a certain compromise. Therefore, it is essential to determine the critical loading rate of the rigid plate to achieve a reasonable quasi-static loading condition with satisfactory accuracy and computational efficiency. Subsequently, with the optimized loading rate, cases with three interface property distributions were investigated to quantify their influence on the load–strain curves, crack events, and energy dissipation.

## 2. DE Model Configurations

**Figure 1:**
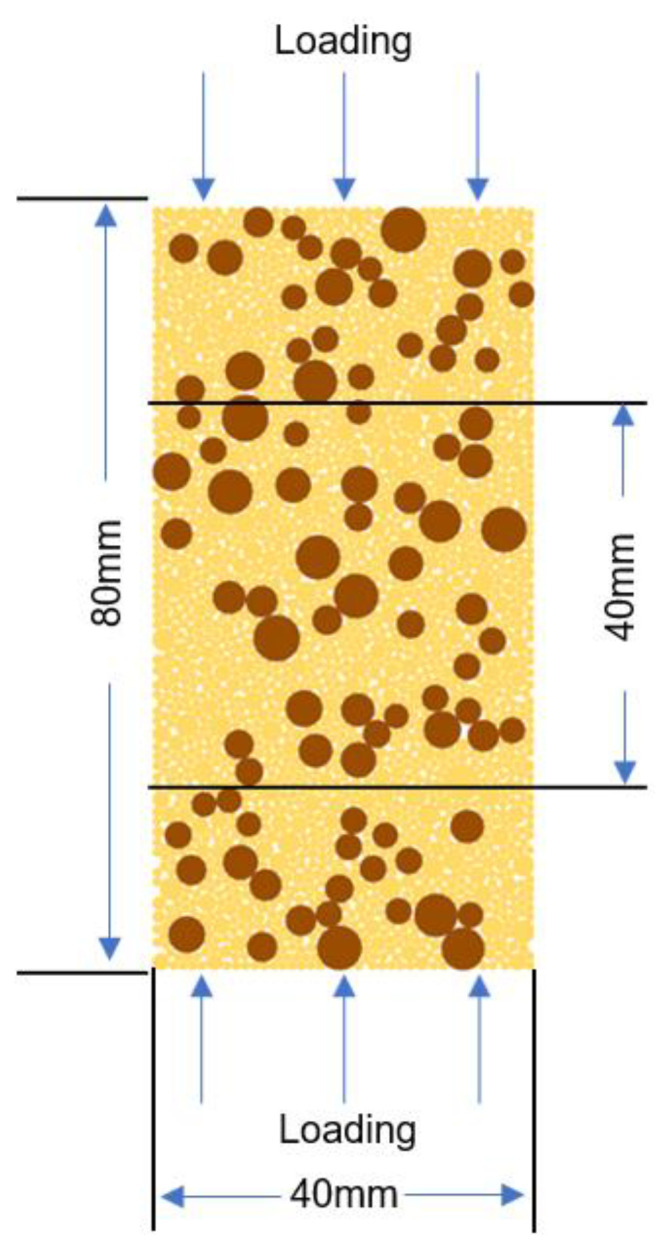
Two-dimensional representation of cold crushing test simulations [23].

A two-dimensional (2D) geometry of 80 mm × 40 mm was defined for the cold crushing test simulations, as shown in Figure 1. In order to decrease the influence of interface friction between the piston and the end surface of samples, the height/diameter ratio of 2 was applied in the simulations [29]. The grain grading of a refractory ceramic material was defined using the Dinger–Funk equation (Equation (1)) [30], with n equal to 0.37, where the maximum and minimum diameter of particles was 5 and 0.088 mm, respectively.

(1) $$P_{d}=\frac{d^{0.37}-d_{min}^{0.37}}{d_{max}^{0.37}-d_{min}^{0.37}}\;,$$

where *P_d_* denotes the cumulative volume distribution of particles with diameters less than *d*. The particle size was divided into 5 ranges: 0.088–1, 1–2, 2–3, 3–4, and 4–5 mm. In each group, the particle size was distributed uniformly.

**Figure 2:**
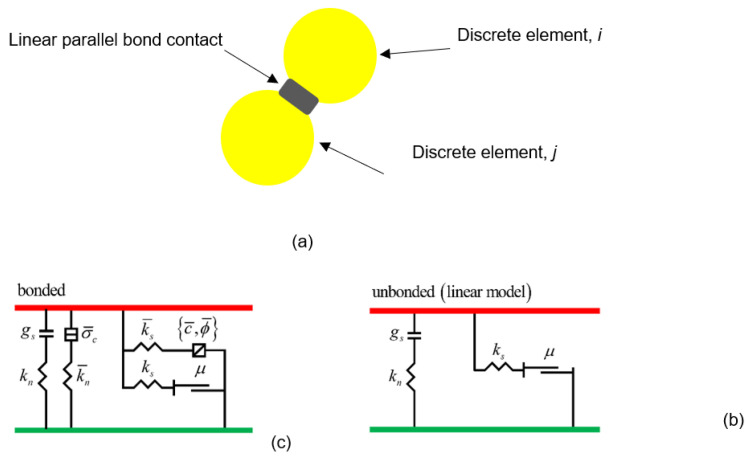
Illustration of linear parallel bond contacts in PFC [31]. (**a**) Parallel bond contact between two circular elements, (**b**) bonded interface law, (**c**) unbonded interface law.

The interface properties between elements were defined using the linear parallel bond model in the Particle flow code (PFC) [31]. The linear parallel bond model (Figure 2) describes the contact between two pieces with two artificial interfaces: unbonded and bonded interfaces. The unbonded interface cannot resist tension and relative rotation; a Coulomb limit is imposed on the shear force, which allows slipping. The bonded interface can resist tension and relative rotation and behaves linearly before the strength limit is exceeded. In the case of bond breakage, the second interface cannot carry the load, and the unbonded interface plays a vital role; when the bond is intact, the two interfaces are active. The terms *g_s_*, *μ*, *k_n_*, and *k_s_* denote the surface gap, friction coefficient, normal stiffness, and shear stiffness of the unbonded interface, respectively; $\bar{\sigma }$*_c_*, $\bar{c}$, $\bar{\phi }$, $\bar{k}$*_n_*, and $\bar{k}$*_s_* denote the tensile strength, cohesion, friction angle, normal stiffness, and shear stiffness of the bonded interface, respectively.

An alternative method to define the interface properties is to define the effective moduli and stiffness ratio instead of stiffnesses, as expressed in Equations (2) and (3).

(2) $$\{\begin{array}{c} \;k_{n}=AE^{*}/L\;,\;unbonded\;\\ {\bar{k}}_{n}={\bar{E}}^{*}/L\;,\;bonded\; \end{array},\;$$

(3) $$\{\begin{array}{c} \;k_{s}=k_{n}/k^{*},\;unbonded\\ \;{\bar{k}}_{s}={\bar{k}}_{n}/{\bar{k}}^{*},\;bonded \end{array},\;$$

(4) A=2rt, 2D (t=1), 

(5) $$r=\{\begin{array}{c} \min\left(R^{\left(1\right)},R^{\left(2\right)}\right),\;ball-ball\;\\ R^{\left(1\right)},\;ball-facet \end{array}\;,\;$$

(6) $$L=\{\begin{array}{c} \;R^{\left(1\right)}+R^{\left(2\right)},\;ball-ball\\ R^{\left(1\right)},\;ball-facet \end{array},\;$$

where E* and ${\bar{E}}^{*}\;$ denote the effective moduli for the unbonded and bonded interfaces, respectively; k* and ${\bar{k}}^{*}$ are normal to shear stiffness ratios of the unbonded and bonded interfaces, respectively; *A* is the contact area; *r* is the radius of the elements; *L* is the distance of the contacted elements; and *R*^(1)^ and *R*^(2)^ are the radii of the contacted elements.

In order to determine a reasonable parameter set for the linear parallel bond model, a brittle refractory ceramic material was used for wedge splitting [32] and cold crushing tests in the laboratory. Subsequently, an inverse identification procedure utilizing the NL2SOL algorithm [33] was applied to fit the experimental and simulation curves from 2D DE models with homogenous interface properties by comparing the experimental and simulated ascending parts and peaks of the curves.

**Figure 3:**
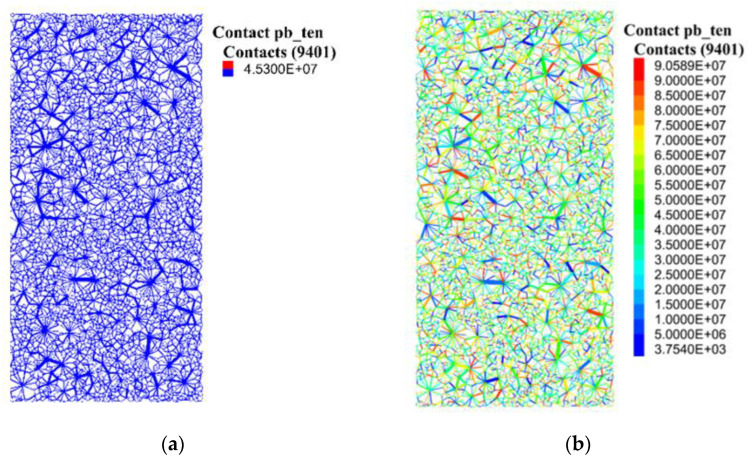

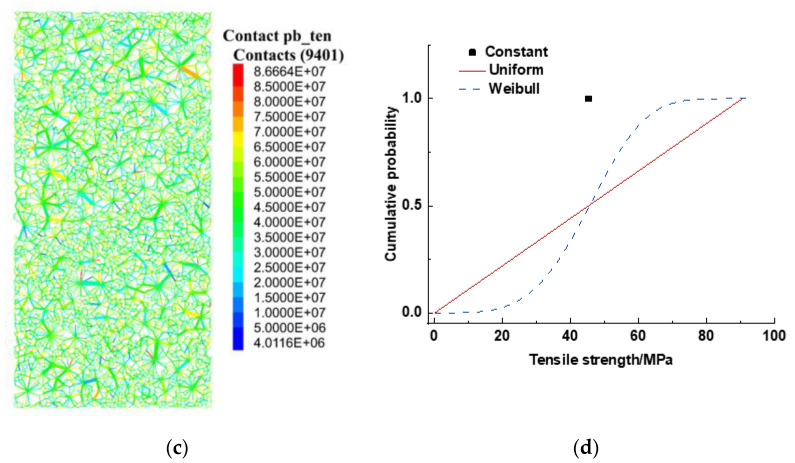
(**a**) Constant, (**b**) uniform, and (**c**) Weibull distributions of tensile strength in the models and (**d**) the tensile strength cumulative curves of the three cases.

Table 1 lists the inversely identified parameters of a homogenous interface, which served as the reference case in this study. Two additional statistical distributions, namely the Weibull and uniform distributions, were considered. The median value of each parameter in the cases with uniform and Weibull distributions was equal to the value of the corresponding parameter of the reference case. An example is shown in Figure 3. The constant distributed parallel bond tensile strength is illustrated in Figure 3a, and the uniformly and Weibull distributed parallel bond tensile strengths are illustrated in Figure 3b,c, respectively. The distribution curves of the tensile strength for the three cases are shown in Figure 3d. Table 2 lists the identified parameters of the contacts between the particles and pistons, which were set to be constant for all three cases.

The load was applied in the vertical direction, as shown in Figure 1. In the laboratory tests, the loading displacement rate was 0.5 mm/min, which was equivalent to a loading strain rate of 1.04 × 10^−4^ s^−1^ with the present geometry. Applying a practical loading strain rate requires substantial computation time, and an increased loading rate for the simulation is desirable. Therefore, different loading strain rates were applied to the simulation of the reference case, and the results were compared. Finally, an optimized loading strain rate was selected to compromise the quasi-static condition and computation efficiency.

## 3. Results and Discussion

### 3.1. Optimized Loading Strain Rate

For a given DE model, the critical time step is determined by the masses, moments of inertia, and stiffness of the elements [34]. In order to obtain a stable calculation, an automatic timestep determination option was applied, which yielded a timestep of 5.5 × 10^−9^ s for the reference case. With limited computational resources, simulation using the practical loading strain rate is highly expensive when obtaining a load–displacement curve, including a major part of the post–peak curve.

**Figure 4:**
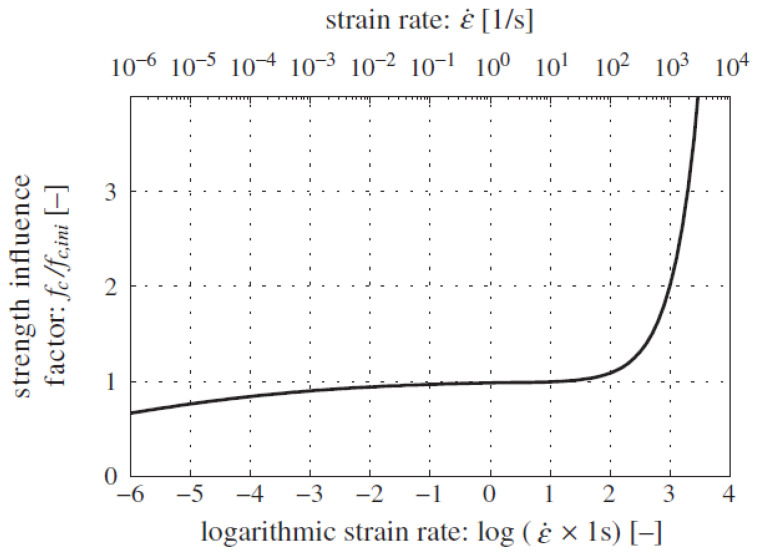
Factors influencing strength as a function of strain rate for a cement paste [36].

**Figure 5:**
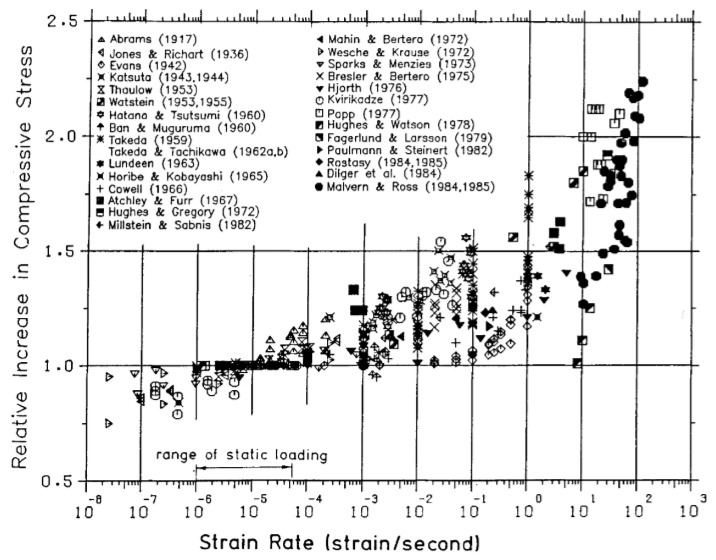
Influence of the strain rate on the compressive strength of concretes [37,38,39,40,41,42,43,44,45,46,47,48,49,50,51,52,53,54,55,56,57,58,59,60,61,62,63,64,65,66].

With an increased loading strain rate, the elapsed computation time of the DE simulation was reduced. Nevertheless, one shall consider that the strength, critical failure strain, and energy dissipation of materials may vary with the loading rate. Therefore, a quasi-static condition is guaranteed for the cold-crushing test simulation. Field et al. [35] presented a schematic of the range of strain rates with respect to different loading conditions, wherein a strain rate range of 10^−4^–10^0^ s^−1^ was treated as quasi-static, and the inertia effect can be negligible. Fisher et al. [36] demonstrated that the compressive strength of cement paste remained unchanged until the loading rate was higher than 10^2^ s^−1^, as shown in Figure 4. Bischoff and Perry [37] conducted a literature survey on the compressive behavior of concrete at high strain rates ranging from 10^−8^ to 10^2^ s^−1^, as shown in Figure 5. They demonstrated that a typical range of static loading ranged from 10^−6^ to 6 × 10^−4^ s^−1^, and the definition of the quasi-static loading range varied with respect to materials and testing conditions. Thus, quantifying the influence of the loading strain rate on the computational efficiency, stress–displacement curve, and crack propagation in the cold crushing test simulation of refractories is necessary.

**Figure 6:**
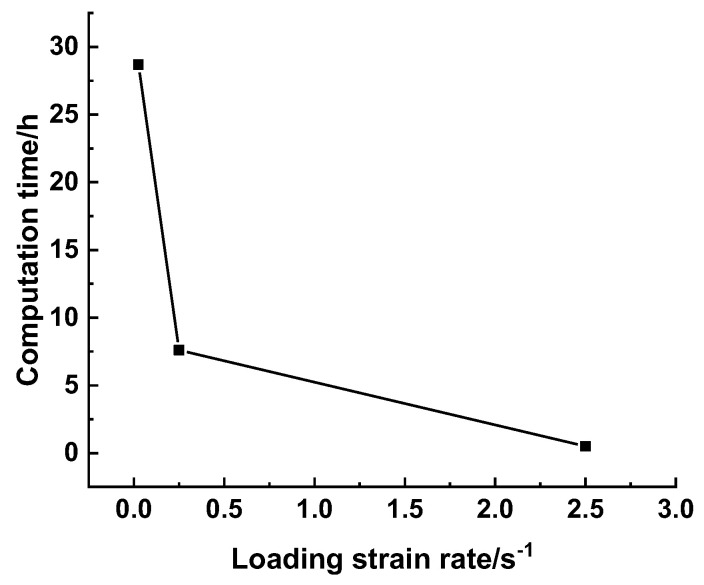
Influence of the loading strain rate on the elapsed time.

The results (Figure 6) revealed that the time consumed (using a workstation equipped with an Intel X5647 CPU and 64 GB RAM) for the post–peak load to achieve 15% peak load increased from 0.5 to 7.6 h as the loading rate decreased from 0.25 s^−1^ to 0.025 s^−1^. With a further decrease in the loading strain rate to 0.025 s^−1^, the elapsed time increased to 28.7 h.

**Figure 7:**
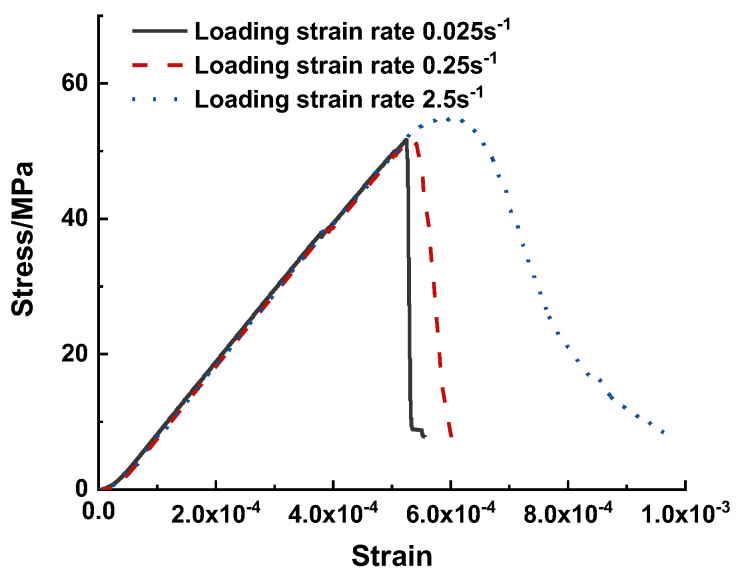
Stress–strain curves of cases with varying loading strain rates.

The stress–strain curves obtained from the three loading strain rates are shown in Figure 7. The strain was recorded on the loading walls. The cold crushing strength and fracture energy were determined using Equations (7) and (8).

(7) $$\sigma_{ccs}=\frac{F_{max}}{A_{0}},$$

where *σ_ccs_* denotes the cold crushing strength, *F_max_* is the maximum force, and *A*_0_ is the cross-sectional surface area.

(8) $$G_{f}=\frac{1}{A_{0}}\int_{0}^{\delta_{ult}}Fd\delta ,\;$$

where *G_f_* denotes the specific fracture energy, *δ_ult_* is the ultimate displacement, and *F* and *δ* are the force and displacement, respectively.

Figure 7 also revealed that the post–peak curve became milder when the loading strain rate increased, and the ultimate strain at 15% post–peak stress increased with respect to the loading rate. The cold-crushing strengths and fracture energies for the three cases are summarized in Table 3. The cold crushing strength was 51.65, 52.17, and 54.91 MPa, whereas the fracture energies were 1098.00, 1246.50, and 2200.25 N/m for the cases with loading strain rates of 0.025, 0.25, and 2.5 s^−1^, respectively. The cold crushing strengths and the fracture energies of the case with a loading strain rate of 0.25 s^−1^ were 1.01 and 1.14 times that of the reference case, respectively. For the case with a loading strain rate of 2.5 s^−1^, the cold crushing strength was 1.06 times that of the reference case; however, the fracture energy was two times that of the reference case. The higher cold crushing strength and fracture energy observed in this study are attributed to the lateral inertial confinement [67].

The number of contacts and cracks were recorded during the simulations, and the crack density was determined as expressed in Equation (9), as follows.

(9) $$\rho_{c}=\frac{n}{N},$$

where *ρ_c_* denotes the crack density, *n* is the number of cracks, and *N* is the number of contacts.

**Figure 8:**
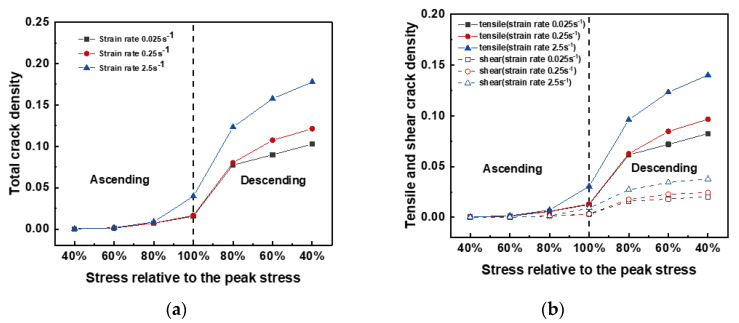
Crack density vs. stress relative to the peak stress for the cases with different loading rates. (**a**) Total crack density, (**b**) tensile and shear crack density.

The crack densities are shown in Figure 8; here, the abscissa represents the ratio of the stress at a certain displacement to the cold crushing strength of the corresponding case. The crack densities began to increase after peak stress of 60% was reached. A monotonic increase in the total crack density was observed during the entire testing stage (Figure 8a). The trend in the crack density increment differed with the loading strain rate. A significant increment occurred after the pre-peak load reached 80% of the peak load and before the post–peak load reached 80% of the peak load for the case with a loading strain rate of 2.5 s^−1^. For the cases with loading rates of 0.25 and 0.025 s^−1^, a significant increment occurred between the peak stress and the post–peak stress approaching 80% peak stress. The total crack density was 0.1776, 0.1213, and 0.1028 for the cases with a loading rate of 2.5 s^−1^, 0.25 s^−1^, and 0.025 s^−1^ at the post–peak part of the 40% peak load, respectively.

Crack events were cataloged as cracks caused by tension and shear. The respective crack densities against the relative stress to the peak stress are shown in Figure 8b. In general, the shear crack density was less than the tensile crack density, and this tendency was observed in all the cases. The tensile/shear crack density increased with increasing loading strain rates. The relative difference between the cases with loading rates of 0.025 and 0.25 s^−1^ was 19.90 and 17.16% for the shear and tensile crack densities, respectively. The relative total crack density variance between the cases with loading rates of 0.025 and 0.25 s^−1^ was 18.01% for the post–peak part of the 40% peak load.

A dimensionless number, the inertial number, is often used to quantify the loading condition, which is expressed as follows [68].

(10) $$I={\dot{\varepsilon }}_{a\;}*d_{max}\sqrt{\frac{\rho }{p}},$$

where ${\dot{\varepsilon }}_{a}$ denotes the axial strain rate, *d_max_* is the maximum diameter of the grains, *ρ* is the density of the grains, and *p* is the mean stress.

Note that usually, a small value of *I* (<10^−3^) signifies quasi-static flow, and a higher value of *I* (>10^−3^) indicates collisional flow with a profound effect of particle inertia [69,70,71]. For polydisperse systems, Shire et al. [72] suggested a smaller critical value of *I* (<10^−5^), particularly for those samples whose strain was large during mechanical tests. Modenese et al. [73] considered the inertial number at peak stress as the critical inertial number.

**Figure 9:**
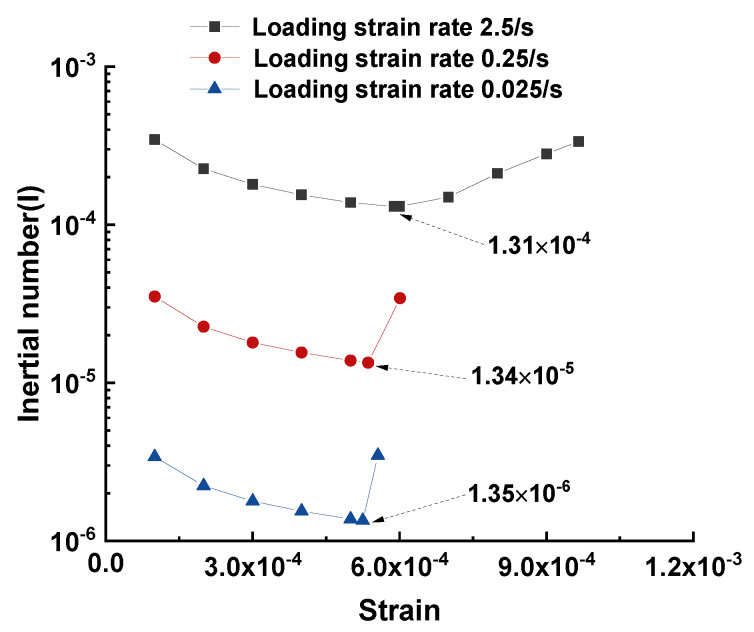
Inertial number with respect to axial strain at varying loading rates ranging from 0.025 s^−1^ to 2.5 s^−1^.

In the 2D simulation of this study, *p* equals $\frac{\sigma_{x}+\sigma_{y}}{2}$, $\sigma_{x}$ denotes the stress in the horizontal direction, and $\sigma_{y}$ denotes the stress in the vertical direction. The particles used in this study had the same density of 3000 kg/m^3^. In order to compare the loading states of the three cases, the largest diameter was used to determine the mass of the particle. The curve of the inertial number against vertical strain is plotted in Figure 9. Evidently, at the beginning of the loading stage, *I* was higher than that at the peak stress, and after peak stress, *I* increased because of the decreasing mean stress. *I* had a magnitude of 10^−4^, 10^−5^, and 10^−6^ in the cases with strain loading rates of 2.5, 0.25, and 0.025 s^−1^, respectively.

The following conclusions could be drawn for the case with a loading strain rate of 0.25 s^−1^: (1) The inertial number was in the magnitude of 10^−5^, and less than 10^−3^; (2) its cold crushing strength variance was negligible compared with that at the loading strain rate of 0.025 s^−1^. These conclusions justify that the loading strain rate of 0.25 s^−1^ for the tested sample can be treated as a quasi-static loading condition.

The DE simulation revealed that under this quasi-static loading condition, the fracture energy and crack events after residual stress reaching 80% peak stress exhibited slightly different quantitative results from those obtained under static loading conditions. It indicates that the system becomes unstable during the latest cracking process. By considering the computation efficiency along with the above conclusions, the loading strain rate of 0.25 s^−1^ was applied in subsequent research activities.

### 3.2. Influence of Interface Properties

#### 3.2.1. Stress–Strain Curves

**Figure 10:**
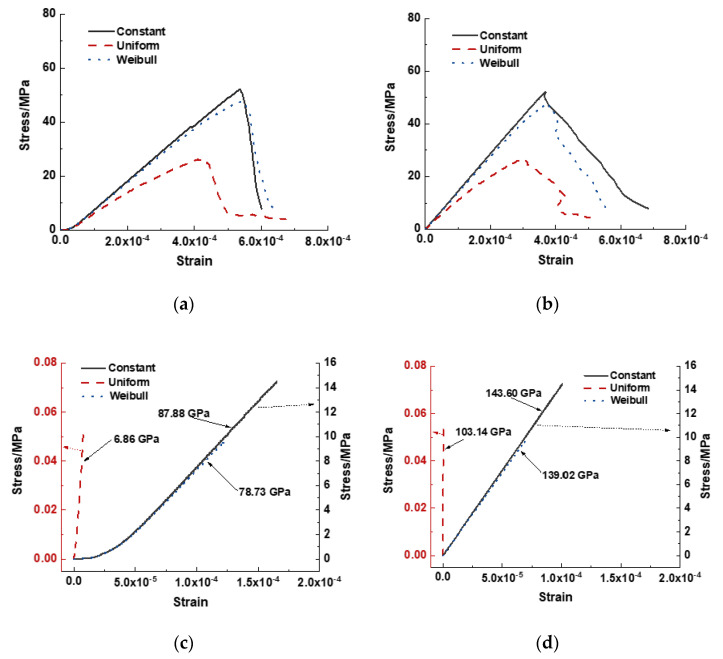
Stress–strain curves of cases with varying interface properties distributions. (**a**) Strain recorded from the relative displacement of loading walls; (**b**) strain recorded from gage particles; (**c**) stress–strain curve recorded from loading walls before the first crack occurs; (**d**) stress–strain curve recorded from gauge particles before the first crack occurs.

The stress–strain curves obtained for the cases with different interface properties are plotted in Figure 10. The strain illustrated in Figure 10a was measured from the relative displacement of the two loading walls, whereas in Figure 10b, it was measured from the relative displacement gauging elements of the specimen, as shown in Figure 1, with an initial distance of 40 mm. The ascending curves of the cases with Weibull distributions overlapped at the beginning of the loading and diverted at higher strains. The case with the Weibull distribution exhibited a crushing strength of 47.73 MPa, whereas the case with constant interface properties exhibited 52.17 MPa. After the peak load, the curves of these two cases decreased significantly. The case with a uniform distribution evidently exhibited a mild slope of ascending and descending parts, with a cold crushing strength of 26.29 MPa.

Similar trends for the three curves are illustrated in Figure 10b. Evidently, the curves recorded from the relative displacement of the gauge elements exhibited a sharp increase at the beginning of the loading procedure, whereas those recorded from the loading wall displacement exhibited only a mild increase. Moreover, the corresponding strain at the peak load shown in Figure 10a was greater than that shown in Figure 10b.

The ascending curves before the cracking of the specimens are plotted in Figure 10c,d for the two displacement recording methods. The case with uniform distribution exhibited cracks at the smallest strain and lowest stress, 7.86 × 10^−6^ and 0.54 MPa, as shown in Figure 10c, and 5.23 × 10^−7^ and 0.54 MPa, as shown in Figure 10d. The secant modulus was determined for the linear part of each curve. Evidently, the curves recorded from the loading wall yielded a less secant modulus than those from the gauge elements. The former included the contribution from the contacts between the positions and end faces of the specimen. The secant value of the case with uniform distribution illustrated in Figure 10d was almost equal to the effective Young’s modulus defined in the interface properties, and the secant value of the other two cases was 1.34 times the effective Young’s modulus.

The fracture energies determined from the curves plotted in Figure 10a,b are listed in Table 4. Different results were observed for cases with the same distribution. This is reasonable because cracks also occurred in the area out of the gauge region. Another factor affecting the determined results is the inconsistent displacement among the gauge elements after post–peak. As shown in Figure 10b, in the case of uniform distribution, a decrease in the axial strain was observed in the descending part at strains ranging from 4.07 × 10^−4^ to 4.32 × 10^−4^. This resulted from the large local displacements of certain elements in the gauge-element group.

#### 3.2.2. Crack Events

**Figure 11:**
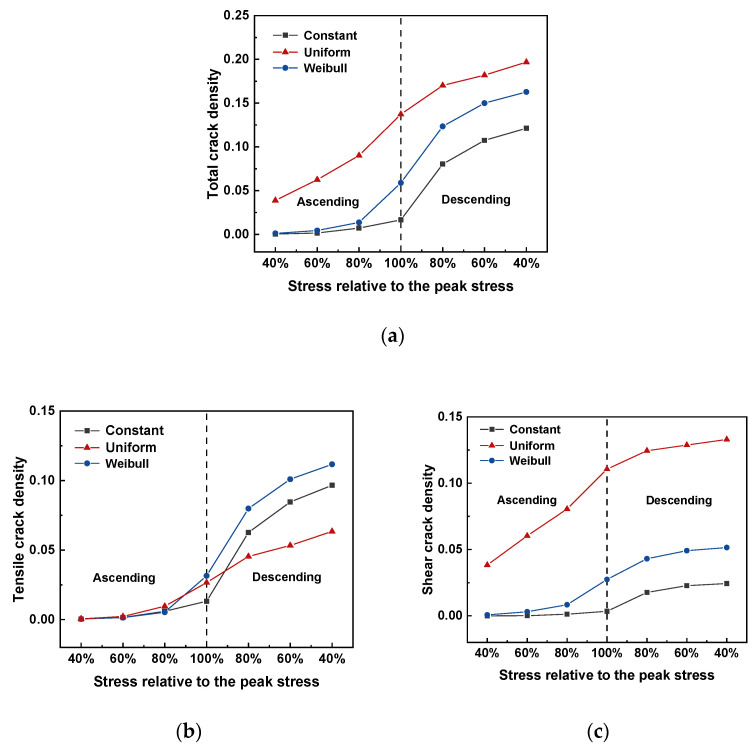
(**a**) Total crack density, (**b**) tensile crack density, and (**c**) shear crack density with respect to stress relative to the peak.

The total crack density evolution against the stress relative to the peak stress differed for cases with different interface property distributions, as shown in Figure 11a. The cracks in the case with a uniform distribution occurred earlier during the testing than those with constant and Weibull-distributed interface properties. For instance, at 40% of peak stress of the ascending curve, the total crack density for the case with a uniform distribution was 0.039, whereas no cracks were observed in the other two simulated samples. The crack density began to increase after 60% peak stress in the case with constant interface properties and after 40% peak stress in the case with the Weibull distribution. Subsequently, a sharp increase in the total crack density occurred between 80% peak stresses before and after the peak load for the case with the Weibull distribution. The case with constant interface properties exhibited a sharp increase between the peak and descending loads at 80% peak stress. After the post–peak curve reached 80% peak stress, the increase in the total crack density became mild for all three cases. The ultimate total crack density of the case with a uniform distribution was the highest among the three cases, followed by the case with the Weibull distribution.

Tensile and shear failures can occur simultaneously during the cold crushing tests. The crack events were divided into two groups, one of which was termed tensile crack density, and the other was shear crack density. Figure 11b,c illustrate the evolution of the respective crack density with respect to the stress relative to the peak stress. Tensile cracks occurred after the load reached 60% peak stress. A sharp increase in the tensile crack density was observed after the peak stress for the cases with constant and Weibull-distributed interface properties. At residual stress equal to 40% peak stress, the case with the Weibull distribution exhibited the highest tensile crack density, whereas the case with uniform distribution exhibited the lowest tensile crack density. The shear crack density of the case with a uniform distribution was higher than that of the other two cases.

**Figure 12:**
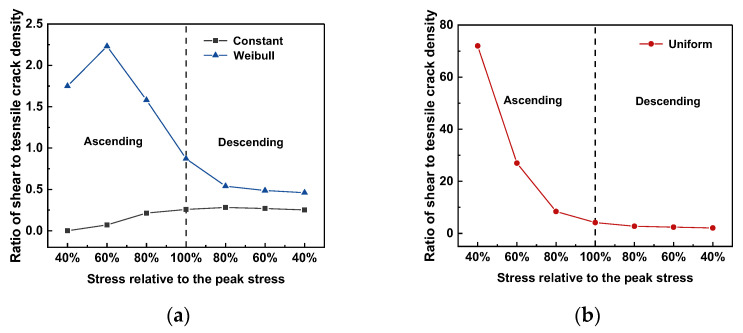
Ratio of crack density of different cases with respect to the stress relative to the peak stress. (**a**) Constant and Weibull distributions; (**b**) uniform distribution.

The ratio of the shear crack density to the tensile crack density relative to the peak stress is shown in Figure 12a,b. A ratio larger than one indicates that shear cracks dominate crack events; when it is less than one, tensile cracks dominate.

Herein, the development of crack density varied with respect to the stress relative to the peak stress. In the case with constant interface properties, a monotonic increase in this ratio was a function of the relative stress between 40 and 80% of the peak stress before the load reached the peak stress; it tended to be a constant value of 0.3. This indicated that tensile cracks dominated the crack propagation in the case with constant interface properties. In the case of Weibull distribution, before the ascending load reached 80% peak stress, the shear crack number was higher than the tensile crack number. Subsequently, the shear-to-tensile crack-density ratio decreased and reached 0.5. In contrast to the case with constant interface properties and similar to the case with the Weibull distribution, the crack density ratio in the case with a uniform distribution decreased monotonically against the stress evolution and reached 2.1.

**Figure 13:**
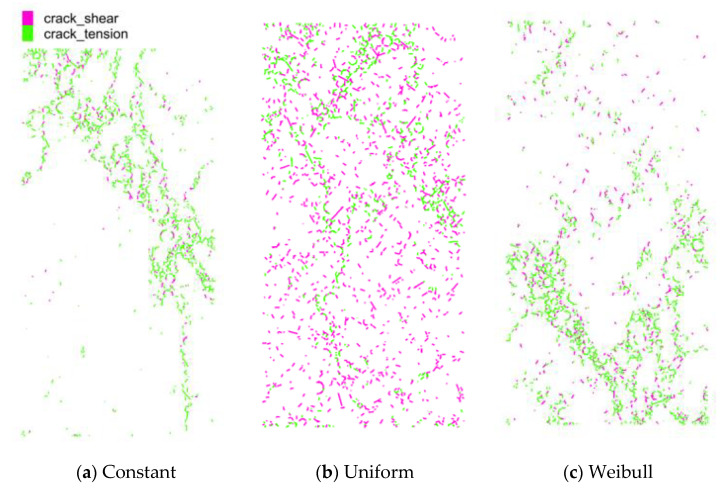
Crack patterns of the cases with different interface properties.

Figure 13 illustrates the ultimate crack patterns for the three cases. Different crack patterns were observed in the simulation for the three cases. The case with constant interface properties exhibited one distinct localized fracture band, which inclined to the end face and passed through more than half the height of the specimen. The case with the Weibull distribution exhibited two major localized fracture bands, which also inclined to the end face and went through approximately half the height of the specimen; the case with a uniform distribution demonstrated dispersive cracks in the entire specimen.

#### 3.2.3. Energy Dissipation

**Figure 14:**
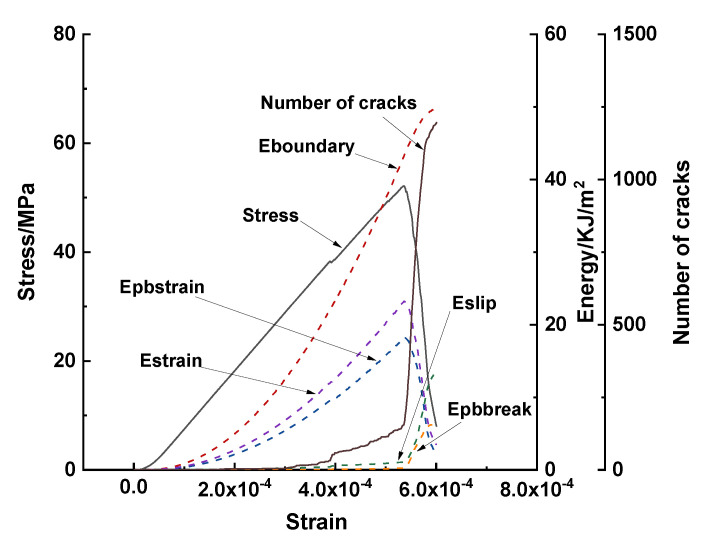
Energy, stress, and crack numbers with respect to strain for the case with constant interface properties.

The total energy dissipation, stress, and crack number for the case with constant interface properties are shown in Figure 14, with respect to the strain recorded from the loading walls. Herein, Eboundary denotes the accumulated energy from the loading walls; Estrain and Epbstrain denote the strain energies stored in the linear and parallel bond springs of the linear parallel bond model, respectively; Eslip denotes the slip energy dissipated by frictional slip; and Epbbreak denotes the bond strain energy at the onset of failure.

The total energy slightly increased with the increase in strain at the beginning of the loading and thereafter rapidly increased before the stress reached the peak load. Subsequently, the total energy continued to increase slightly. The elastic energies exhibited similar trends before the peak load was reached. After the peak load, the elastic energy was released because of the bond damage. This was confirmed by the increase in Eslip and Epbbreak. Crack propagation began at a strain of 2 × 10^−4^ and increased slightly at the beginning. A sharp increase occurred immediately after the peak load, which indicated a crack initiation burst at this stage. The crack number continued to increase, and the crack number increase became mild at the later stage of post–peak curve, which corresponded to a slight increase in Eboundary.

**Figure 15:**
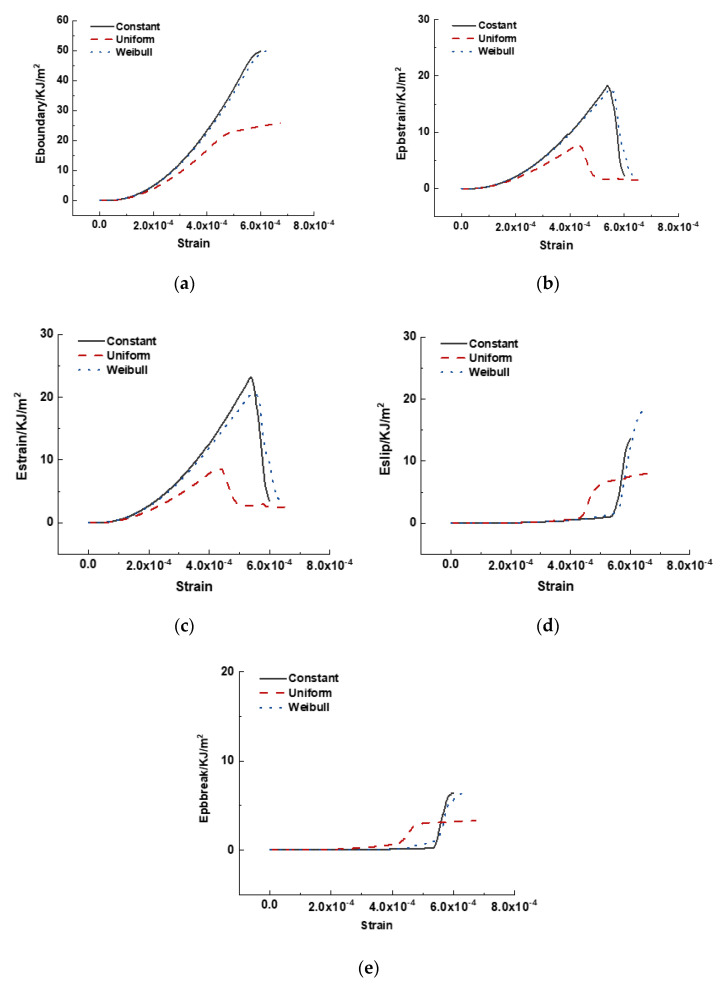
Comparison of energy dissipated by different mechanisms in cases with varying interface properties distribution. (**a**) Eboundary with respect to strain, (**b**) Epbstrain with respect to strain, (**c**) Estrain with respect to strain, (**d**) Eslip with respect to strain, and (**e**) Epbbreak with respect to strain.

A comparison of the different dissipated energies in cases with different interface properties is shown in Figure 15. The total energy consumed in the cases with constant and Weibull distributed interface properties was similar, whereas that in the case with uniformly distributed interface properties was smaller. Additionally, the elastic energies stored in the cases with constant and Weibull-distributed interface properties were similar at the beginning of the ascending part before the strain reached 4.5 × 10^−4^. Subsequently, the elastic energies in the case with Weibull distributed interface properties became smaller than that in the case with constant distributed interface properties. Evidently, the stored elastic energy in the case with uniformly distributed interface properties was the least.

The case with a uniform interface property distribution exhibited the highest Eslip and Epbbreak before the peak load, and the crack appeared much earlier than in the other two cases. The Weibull distributed interface properties case exhibited a higher value of Eslip and Epbbreak after the peak load due to a sharp increase in the crack number and propagation.

## 4. Conclusions

This study presented the results of 2D DE modeling of uniaxial cold crushing tests. The results revealed that the computation efficiency was enhanced significantly when the loading strain rate was 0.25 s^−1^, and a further increase in the loading strain rate to 2.5 s^−1^ resulted in a substantial increase in the dissipated energy and crack numbers. By considering the computation efficiency and quasi-static conditions, the intermediate loading strain rate of 0.25 s^−1^ was applied in subsequent investigations.

The manner of recording the deformation affects the stress–strain curves. The contribution of the loading wall and specimen interface resulted in a low Young’s modulus when the loading wall displacement was recorded. Special cautions shall be given in calculating Young’s modulus out of the cold-crushing tests.

The effects of the interface property distributions on the stress–strain curves and crack events were studied. The uniform distribution resulted in a slight increase in stress with respect to strain during the ascending and early nonlinear shapes before reaching the peak load due to premature failure. In contrast, the Weibull distribution exhibited a distinct linear increase in the ascending part until approximately 80% peak load. The divergence of the ascending parts between cases with constant and Weibull distributed interface properties became distinct after 80% peak load. A significant variance in the crack patterns was observed for the cases with different interface property distributions. These variances among the ascending parts of the stress–strain curves and the crack pattern obtained from the simulations suggest that the distributed material properties can be used to represent the heterogeneity of a specified material. Further information, such as fracture information received from digital image correlation or acoustic techniques, from laboratory tests can be used to determine the distribution parameters.

Additionally, the DE modeling allowed the quantification of crack events and their respective dissipated energies, thereby providing insights into the fracture of refractories. The dominant fracture mechanism and energy dissipation depended on the interface properties and their statistical distributions.

## Figures and Tables

**Table 1:** Parameter set for the linear parallel bond model with constant values.

Interface		Properties	Values
Contacts between particles	Bonded and unbonded	Effective modulus (GPa)	104
		Normal to shear stiffness ratio	1.27
	Unbonded	Friction coefficient	0.493
	Bonded	Friction angle (°)	40
		Cohesion (MPa)	40
		Tensile strength (MPa)	53

**Table 2:** Parameter set for the contacts between particles and pistons.

	Properties	Values
Contacts between pistons and particles	Effective modulus (GPa)	4.8
	Normal to shear stiffness ratio	1.27
	Friction coefficient	0.09

**Table 3:** Cold crushing strengths and fracture energies of the cases with varying loading strain rates.

Loading Strain Rate (s^−1^)	Cold Crushing Strength (MPa)	Relative Cold Crushing Strength	Fracture Energy (N/m)	Relative Fracture Energy
2.5	54.91	1.06	2200.25	2.00
0.25	52.17	1.01	1246.50	1.14
0.025	51.65	1.00	1098.00	1.00

**Table 4:** Dissipated energies determined from the stress–strain curves recorded from loading walls and gauge elements.

Interface Properties Distribution	Dissipated Energy (Walls) (N/m)	
	Loading Walls	Gauge Elements
Constant	1246.50	1449.75
Uniform	646.50	593.00
Weibull	1264.50	1148.50

## Data Availability

Not applicable.
